# Teen Advisory Council Survey's Factors Associated With Self-Harming Thoughts

**DOI:** 10.3389/fpsyt.2022.851477

**Published:** 2022-06-23

**Authors:** Pamela McPherson, Laura Lane Alderman, Jazzlynn Temple, Robert Lawrence, Victor J. Avila-Quintero, Johnette Magner, Caroline E. Sagrera, James C. Patterson, Kevin S. Murnane

**Affiliations:** ^1^Department of Psychiatry and Behavioral Medicine, School of Medicine, Louisiana State University Health Sciences Center—Shreveport, Shreveport, LA, United States; ^2^Louisiana Addiction Research Center, Shreveport, LA, United States; ^3^Caddo Parish Magnet High School, Shreveport, LA, United States; ^4^Yale University, New Haven, CT, United States; ^5^Child Study Center, Yale School of Medicine, New Haven, CT, United States; ^6^School of Communication and Media Studies, Louisiana Tech University, Ruston, LA, United States; ^7^Department of Pharmacology, Toxicology and Neuroscience, School of Graduate Studies, Louisiana State University Health Sciences Center—Shreveport, Shreveport, LA, United States

**Keywords:** self-harming thoughts, suicide, lived experience, stress, Gen Z, Caddo Parish

## Abstract

**Background:**

The evaluation of teens with self-harming thoughts (SHT) is a high-stakes task for physicians in community and emergency department (ED) settings. The lived experience of adolescents with stress and SHT provides an important source of insight for mental health professionals who evaluate and treat teens A snapshot of the lived experience of teens in northwest Louisiana was captured by the Step Forward Teen Advisory Council (TAC) in 2019. The TAC surveyed peers with the goal of identifying common stressors experienced by local teens in order to inform policy and practices in the local school system. The identification of stressors is a critical step in addressing SHT as adolescents who experience life stressors are at increased risk for self-harming thoughts (SHT), a known precursor to self-harm and suicide. Assessing youth for life stressors is a critical element of suicide prevention.

**Methods:**

Local teens queried 5,070 peers attending Caddo Parish schools to better understand the stressors faced by high school students in Northwest Louisiana using a student developed survey. Results were presented to peers at a virtual summit where teens developed action items to reduce stress and presented findings to local leaders. Their efforts ultimately lead to increased supports for students in local schools.

**Results:**

Over half of the teens surveyed reported stressors that negatively impacted their physical or emotional well-being. Students endorsing self-harming thoughts reported an average of 7.82 stressors as compared to 3.47 in peers without SHT. Teens with stressors at both home and school were more likely to experience SHT than teens with stressors in a single location.

**Conclusion:**

The Gen Z students who developed the TAC Survey identified stress as a major concern for teens in Northwest Louisiana. The TAC Survey data aligns local experience with established data regarding the association between stress, depression and SHT. Second, the results highlight the importance of diving deep to identify all stressors when assessing the risk of self-harm. Finally, the lived experience of local teens with SHT provides critical information for professionals to better understand risk for SHT and suicide in our region and beyond.

## Introduction

The evaluation of teens with self-harming thoughts (SHT) is a high-stakes task for physicians in community and emergency department (ED) settings. ED visits related to SHT doubled nationally between 2007 and 2015 (1, 2). Rates of suicidal ideation (SI) increased in March through July 2020 as compared with 2019; however, rates were significant only in March and July (3). During the pandemic, emergency departments have experienced a marked increase in visits by teens related to suspected suicide attempts, with a 50.6% increase for girls and a 3.7% for boys in 2021 compared to 2019 (4).

Self-harming thoughts (SHT), including but not limited to suicide ideation (SI), are a precursor to self-harm and suicide, the second leading cause of death for youth between the ages of 12–18 years (5). Reports of the lifetime prevalence of SI among adolescents range from 9.6 to 39.4% with increased prevalence noted among specific subgroups, including LGBTQ youth and females (6–9). The rate of SI among LGBTQ adolescents exceeds that of peers, with 42% endorsing SI compared to 14% for those non-identifying youth in a study of nearly 5,000 teens (10). Furthermore, the rate of SI for females is twice that of males (11, 12). Despite a slight overall decrease in self-reported suicidal ideation (SI) between 2015 and 2017, SI remains common with a weighted overall prevalence rate of 18.8% among high school students between 1991 and 2017 (13). Alarmingly, Caddo Parish data does not reflect this trend. According to the Caring Communities Youth Survey, SI increased by 19% among 10th graders and a 27% among 12th graders between 2016 and 2020 (14).

The United States Surgeon General's 2021 *Call to Action to Implement the National Strategy for Suicide Prevention* (15) highlights the importance of screening for SHT. While the presence of a mental disorder is up to 90% in persons who die by suicide, adolescence is a time when mental disorders often emerge. This fact supports the importance of screening for symptoms and identifying emerging mental illness in youth with SHT in order to prevent suicide (16). Psychosocial factors contributing to suicide risk include being bullied, child abuse, stressful life events, and suicide contagion (17–20). Teens lived experience with bullying, abuse, and stress were factors that were also included in the Teen Advisory Council's survey.

Assessing SHT requires query into many factors including symptoms of mental illness, risk and protective factors, and psychosocial stressors. Symptoms of mental illness that are correlated with SHT include depressed mood, anxiety, hopelessness, social isolation, eating disorders, and substance use (9, 21–24). Support from family and friends is protective against SHT, especially for girls (25). Lower parent-family connectedness has been reported among sexual minority youth, a population at increased risk for SHT (26). School connectedness has also been identified as a protective factor in reducing suicide risk (27, 28). Beliefs surrounding hel*p-*seeking may moderate the progression of SHT to a suicide attempt (10). The assessment of suicide risk balances symptoms of mental disorders, risk factors, and psychosocial stressors against protective factors to design individualized safety and treatment plans. Knowledge of local trends in SI, youth stress, and youth supports is critical if clinicians are to protect youth.

### Methods

Step Forward is a community organization in Northwest Louisiana working to intentionally address complex problems in the local community. Specifically, they aim to ensure success for every child from cradle to career. To further this goal, the Step Forward Teen Advisory Committee (TAC) was established in the spring of 2019 to allow local teens to share their knowledge and perspectives regarding the needs of local youth. The TAC served as expert advisors to Step Forward community leaders in to identify key focus areas and to develop action plans aimed at improving outcomes for all teens in Northwest Louisiana. These young leaders identified three key objectives: improve teen mental health, increase diverse career training opportunities, and increase youth civic involvement. Improving teen mental health became the primary focus of the TAC.

With the goal of formulating a strategic plan to improve teen mental health, the TAC first sought to establish a baseline for Teen Mental Wellness in the region through a survey. The TAC survey consisted of 15 questions concerning students' age, gender, parish of residence, and stressors that they may have faced or were facing at the time. Additional questions focused on feelings of belonging, engagement with their school and community, and social media usage. The survey questions were reviewed by a professional mental health evaluator for scope and language use, specifically to focus on wellness rather than mental illness.

The local school superintendent approved survey before it was distributed through an online platform, SurveyMonkey. All students in attending public high schools in Northwest Louisiana were invited to participate individually and during their English classes from mid-December 2019 to mid-January 2020. The survey received 5,070 student responses with student ages ranging from 14 to 19 years. While all high school students were invited to participate, most respondents (4,989) were from the Caddo Parish School District due to the robust support from the superintendent. The data received was first analyzed through SurveyMonkey and then transferred to Microsoft Excel for further analysis by the TAC for presentation to area leaders. Analysis for this article was performed using STATA/BE v17 (StataCorp, LLC). Continuous variables are presented as mean (SD). Categorical variables are presented as the number (proportion or %) of participants. *P-*values for continuous variables correspond to Two-sample *T*-test (for comparison of means between groups). *P-*values for categorical variables correspond to Pearson's χ2 tests. Univariate logistic regression models were used to identify predictors to estimate the odds of the outcome of student variables in relation to SHT.

During the 2019–20 school year, there were 11,248 students enrolled in ten public high schools in Caddo Parish. The demographics of Caddo Parish at this time were characterized by 71% minority populations with 29% being White. 49.52% were female and 50.48% were male with 69.9% considered economically disadvantaged. Population per grade was 9th−3,156; 10th−2,802; 11th−2,666; 12th−2,624.

## Results

### Participants

Respondents to the Teen Mental Wellness Survey included 5,070 Louisiana public high school students in Bossier, Caddo, and Red River parishes, with 98.4 percent of respondents residing in Caddo Parish. The average age for respondents was 15.79 years. Female students were more prevalent in this sample (54.3%) followed by males (43.5%), “Other gender” (1.3%), and “Prefer Not to Say” (1%) (Table 1).

**Table 1:** Table of characteristics stratified by self-harm thoughts.

	**Total Sample**	**No**	**Yes**	***p-*value**
	***N =* 3,640**	***N =* 3,134**	***N =* 506**	
Age (years), mean (SD)	15.78 (1.22)	15.79 (1.22)	15.73 (1.22)	0.27
Gender				<0.001
Other	60 (1.6%)	38 (1.2%)	22 (4.3%)	
Female	2,161 (59.4%)	1,780 (56.8%)	381 (75.3%)	
Male	1,374 (37.7%)	1,284 (41.0%)	90 (17.8%)	
Prefer not to say	45 (1.2%)	32 (1.0%)	13 (2.6%)	
**Stressors faced by students**				
Anxiety				<0.001
No	1,727 (47.4%)	1,641 (52.4%)	86 (17.0%)	
Yes	1,913 (52.6%)	1,493 (47.6%)	420 (83.0%)	
Depression or Extreme Sadness				<0.001
No	2,051 (56.3%)	1,988 (63.4%)	63 (12.5%)	
Yes	1,589 (43.7%)	1,146 (36.6%)	443 (87.5%)	
Hallucinations				<0.001
No	3,488 (95.8%)	3,048 (97.3%)	440 (87.0%)	
Yes	152 (4.2%)	86 (2.7%)	66 (13.0%)	
Loneliness				<0.001
No	2,243 (61.6%)	2,102 (67.1%)	141 (27.9%)	
Yes	1,397 (38.4%)	1,032 (32.9%)	365 (72.1%)	
Mood swings				<0.001
No	2,026 (55.7%)	1,881 (60.0%)	145 (28.7%)	
Yes	1,614 (44.3%)	1,253 (40.0%)	361 (71.3%)	
Stress				<0.001
No	1,048 (28.8%)	992 (31.7%)	56 (11.1%)	
Yes	2,592 (71.2%)	2,142 (68.3%)	450 (88.9%)	
Number of stressor types, mean (SD)	4.08 (3.00)	3.47 (2.28)	7.82 (4.04)	<0.001
**Location of personal stressors**				
Your Home				<0.001
No	1,153 (31.7%)	1,083 (34.6%)	70 (13.8%)	
Yes	2,453 (67.4%)	2,018 (64.4%)	435 (86.0%)	
Missing values	34 (0.9%)	33 (1.1%)	1 (0.2%)	
School				<0.001
No	613 (16.8%)	563 (18.0%)	50 (9.9%)	
Yes	2,993 (82.2%)	2,538 (81.0%)	455 (89.9%)	
Missing values	34 (0.9%)	33 (1.1%)	1 (0.2%)	
**Person who students confided with**				
Parent				<0.001
No	1,170 (32.1%)	933 (29.8%)	237 (46.8%)	
Yes	1,134 (31.2%)	999 (31.9%)	135 (26.7%)	
Missing values	1,336 (36.7%)	1,202 (38.4%)	134 (26.5%)	
Teacher				<0.001
No	2,046 (56.2%)	1,739 (55.5%)	307 (60.7%)	
Yes	258 (7.1%)	193 (6.2%)	65 (12.8%)	
Missing values	1336 (36.7%)	1,202 (38.4%)	134 (26.5%)	
School counselor				<0.001
No	2,089 (57.4%)	1,792 (57.2%)	297 (58.7%)	
Yes	215 (5.9%)	140 (4.5%)	75 (14.8%)	
Missing values	1,336 (36.7%)	1,202 (38.4%)	134 (26.5%)	
Things would not get better				<0.001
No	1,426 (39.2%)	1,303 (41.6%)	123 (24.3%)	
Yes	878 (24.1%)	629 (20.1%)	249 (49.2%)	
Missing values	1,336 (36.7%)	1,202 (38.4%)	134 (26.5%)	
Was confiding in someone beneficial?				<0.001
No	506 (13.9%)	375 (12.0%)	131 (25.9%)	
Yes	1,798 (49.4%)	1,557 (49.7%)	241 (47.6%)	
Missing values	1,336 (36.7%)	1,202 (38.4%)	134 (26.5%)	
Engagement in school, extracurricular, or community activities				0.026
No	1,040 (28.6%)	873 (27.9%)	167 (33.0%)	
Yes	2,477 (68.0%)	2,150 (68.6%)	327 (64.6%)	
Missing values	123 (3.4%)	111 (3.5%)	12 (2.4%)	

### Self-Harm by Gender

Some genders were more likely to endorse SHT as a stressor. Female (OR = 3.05; 95% CI = 2.40–3.88), Other Gender (OR 8.26; CI 4.69 to 14.56), and Prefer Not to Say Gender (OR = 5.80; 95% CI = 2.94–11.43) students had a substantially increased risk of endorsing SH as a stressor (Table 2).

**Table 2:** Odds ratio for reporting self-harm thoughts.

	**OR**	**95% CI**	***P-*value**
**By gender**			
Male (Ref)	1	–	–
Female	3.05	2.40 to 3.88	<0.001
Other	8.26	4.69 to 14.56	<0.001
Prefer not to say	5.80	2.94 to 11.43	<0.001
**By location of personal stressors**			
Not at School or Home (Ref)	1	–	–
Only at School	0.88	0.49 to 1.59	0.677
Only at Home	1.39	0.74 to 2.60	0.306
Both at School and Home	3.36	1.97 to 5.74	<0.001
Engagement in school, extracurricular, or community activities	0.79	0.63 to 1.00	0.05

### Stressors Faced by Students With SHT

**Figure 1:**
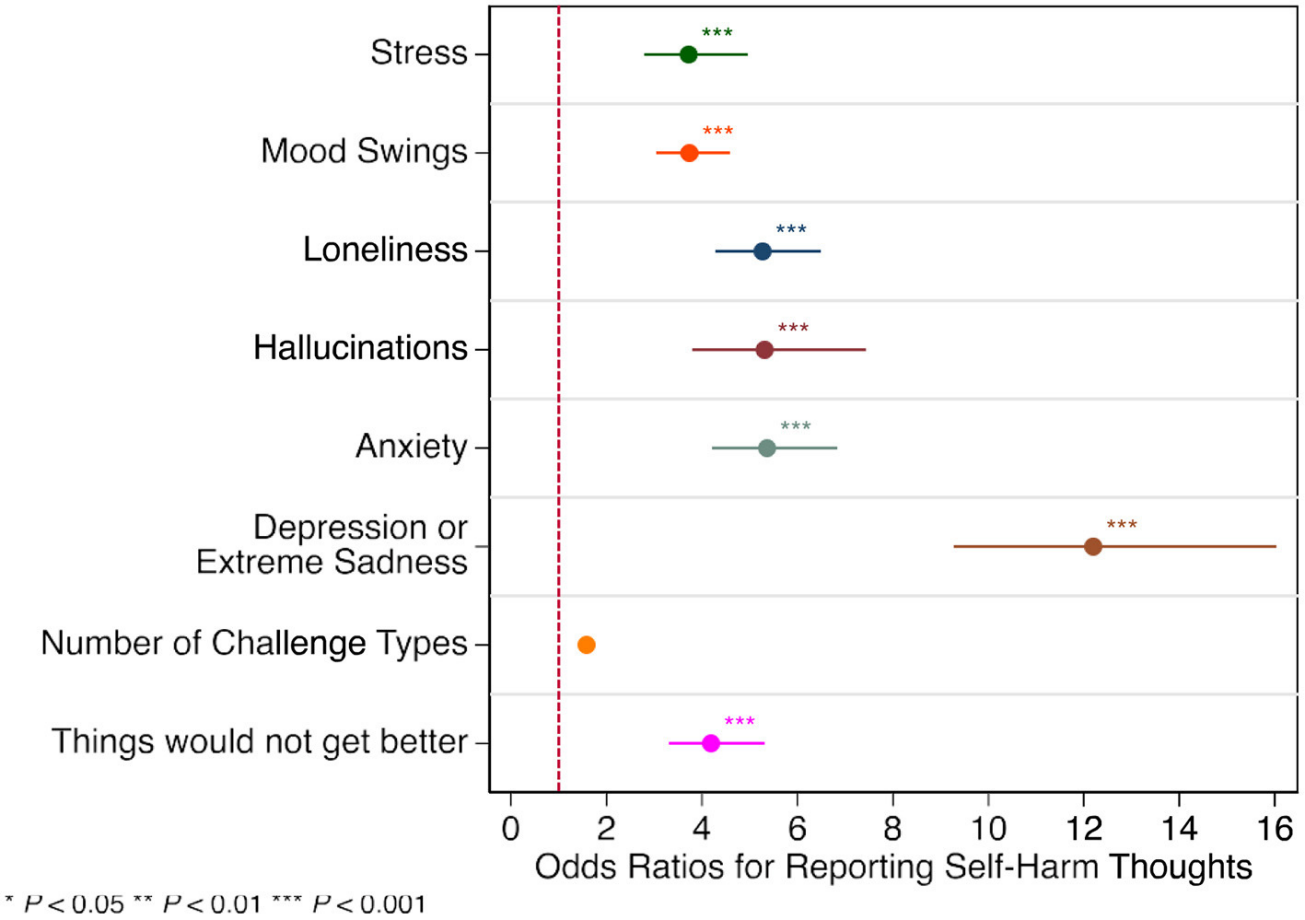
Odds ratios for students reporting self-harm thoughts by stressor type. *P < 0.05, **P < 0.01, ***P < 0.001.

Students who endorsed SHT as a stressor also were at much greater risk for other stressors, including Stress (OR = 3.72; 95% CI = 2.79–4.96), Mood Swings (OR = 3.74; 95% CI = 3.04–4.59), Loneliness (OR = 5.27; 95% CI = 4.28–6.49), Hallucinations (OR = 5.32; 95% CI = 3.80 to 7.44), and Anxiety (OR = 5.37; 95% CI = 4.21–6.84). The stressor with the greatest co-occurring risk for SHT was Depression or Extreme Sadness (OR = 12.20; 95% CI = 9.28–16.04). The average number of stressors was also significantly different revealing that SH was included in an average total of 7.82 stressors as compared to 3.47 (OR = 1.58; 95% CI 1.52–1.65) total stressors for students who did not endorse SHT. Students endorsing SHT were also significantly more likely to believe that things would not get better (OR = 4.19; 95% CI = 3.31–5.31) (Figure 1).

### Location of Personal Stressors

Location of personal stressors, whether home or school, did not reveal a significant difference for students with the stressor of SHT. However, when stressors occurred in both places simultaneously, the risk for SHT was substantially greater (OR = 3.36; 95% CI = 1.97–5.74) (Table 2).

### Other Factors

Students with the stressor of SHT had an associated risk for loneliness. Engagement in school, extracurricular, or community activities was significantly associated with a decreased likelihood of SHT (OR = 0.79; 95% CI= 0.62 to 1; *p* = 0.05) (Table 2). There were also no significant differences in the groups for the perceived benefits of confiding in a teacher or school counselor; however, those who endorsed SHT were more likely consider confiding in a parent to be beneficial (OR = 1.40; 95% CI = 0.89–2.20) (Table 3).

**Table 3:** Odds ratios for finding confiding in someone beneficial among students who reported self-harm thoughts.

	**OR**	**95% CI**	***P-*value**
Confiding in Teacher	1.17	0.6–2.07	0.589
Confiding in School Counselor	1.03	0.61–1.75	0.911
Confiding in Parents	1.40	0.89–2.20	0.141

## Discussion

During patient care, the assessment of suicide risk is one of the most complex and challenging tasks faced by clinicians. Even the most experienced psychiatrists may long for a definitive assessment scale or additional patient information. In examining the lived experience of local high school students, this study provides a valuable additional data source. Namely, the importance of an exhaustive exploration of all stressors faced by the patient. During evaluations clinicians typically identify the most pressing stressor but it is the total stressor load that contributes to self-harm risk, particularly if stress is significant at both home and school.

The high school students who developed the TAC Survey identified stress as a major concern for teens in Northwest Louisiana. This was an astute choice. The TAC are members of Generation Z, persons born between 1997 and 2010. GenZers report a higher level of stress than other groups, and during the pandemic, GenZers have struggled the most. Fortunately, they are more likely to seek mental health services than others. As digital natives, they are at ease with online research, surveys, virtual meetings, and therapy. Many GenZers, like the TAC, are comfortable engaging leaders and leveraging data to promote change. The TAC embraced the concept that all health is local (29) and became influencers for change in our region, documenting their lived experiences and increasing awareness and access to mental health services in our schools.

While the TAC had systems change as a goal, their research yields important lessons for mental health professionals in three major ways. First, the TAC data confirms that our local experience aligns with established data indicating that depression and anxiety are associated with self-harm. Second, the importance of diving deep to identify stressors when assessing youth for suicide prevention is highlighted as ameliorating stressors is critical to minimizing the risk of self-harm. And finally, the experience of local teens with SHT provides critical information for professionals to understand risk in our area and beyond. This is critical knowledge given that SHT and suicide by peers can promote contagion. While this study represents the lived experience of urban and rural teens in the southern United States, similar stresses are widely reported for adolescents, suggesting that the results found here would generalize to other locales (29).

The TAC data shows that symptoms of depression, anxiety, mood swings, and eating disorders are associated with SHT. The TAC study found young women to be at three times the risk for SHT compared young men, in contrast to the findings of Howarth et al. (19) that males were at greater risk than females. This highlights the fact that the assessment of suicide risk is informed by research but ultimately treatment decisions must be based on the presentation and needs of the individual patient. The self-report of symptoms of mental disorders by teens highlights the need for accessible and comprehensive mental health services. In fact, the TAC requested such from local governing bodies, including Bossier Parish Public Schools and Caddo Parish Public Schools. TAC provided the school districts with a list of policy recommendations, including increased interaction with school counselors, incorporating mental health content into health education classes, and education for teachers on the signs and symptoms of mental health disorders in youth. In addition to stress due to symptoms of mental illness, the TAC survey revealed that where stressors occur, the number of stressors is a significant predictor of SHT. A 2020 systematic review and meta-analysis found suicidal ideations were increased by 45% among persons reporting life stressors (19). The TAC data shows over three times the risk for SHT when stress is high at home and school compared to stress in a single setting. This highlights the importance of exploring life circumstances at home and school when assessing youth for SHT. Youth with SHT reported a higher number of stressors than those without SHT, with ranges of 5–11 and 3–5, respectively. This serves as a lesson to mental health professionals to seek to identify as many stressors as possible in order to construct the most beneficial safety plan possible by addressing each stressor specifically and identifying where the teen feels safe. If stressors are present in both school and home, the lack of a safe respite from one or the other may result in unrelenting distress during all waking hours. Life events scales can assist in identifying stressors, but a follow-up interview is necessary to understand the meaning of the stressful event to the adolescent in order to intervene appropriately (30).

### Potential Limitations

This study is limited due to the use of a non-validated, self-report survey instrument. A standardized instrument would have resulted in greater generalizability and would have allowed more robust comparison to existing data on stress in teens. The TAC Survey was created by the TAC to promote system change rather than measure variables associated with SHT. It was not designed to be a scientific research instrument. By design, the instrument did not capture race, sexual orientation, or gender identity—all critical factors for understanding SHT and preventing suicide. Finally, this study examines the lived experience of American teens in northwest Louisiana, the generalizability to broader populations, while likely, is uncertain and bears further study.

### Future Directions

The TAC Survey data identified the importance of comprehensive exploration of stressors as part of the overall assessment of SHT and Safety Planning. Each stressor represents a critical intervention point for suicide prevention. This finding has important implications for safety planning and the design of safety planning forms. The Warning Signs section of most Safety Plan templates captures stressors but does not encourage listing more than a few warning signs and does not explicitly indicate that location where the stressor is experienced should be documented (31). Constructing a Safety Plan in collaboration with the patient and family is the current standard of care. This study suggests that the failure to explore all stressors contributing to distress and hopelessness represents a missed opportunity for preventing death by suicide. Future research might explore the feasibility and benefits of expanding the Warnings Signs section when completing a collaborative Safety Plan with adolescents to capture 5–7 stressors that are warning signs. In this study, students with SHT experienced an average of 7.8 stressors. While understanding the most significant stressor is important, reducing the number of stressors could be just as beneficial and remains an area for future study.

The TAC Survey found that stressors in multiple locations can increase risk for SHT, highlighting the importance of a safe harbor as part of overall suicide prevention efforts. Providing parents, teachers, coaches, and others working with teens information on how to support teens and reduce stress in order to create a safe place is critical. Student advocacy has led some states and school districts to recognize mental health days as excused absences, creating a brief space free of academic stress (32). The impact of such efforts to reduce SHT is promising but bears further study. In addition to reducing the number of stressors, a therapeutic goal should be to establish a safe haven from distress. Safety planning should include discussion of places where the teen feels safe. Cyberspace, a locus without physical boundaries, as a source of both distress and a safe haven must also be considered. The influences of social media and gaming and teens strategies for recognizing and managing these influences must be understood for a given individual as part of safety planning to manage SHT.

Finally, the use of a teen-developed survey to leverage change across an entire school district with the subsequent presentation of the data at a regional suicide prevention summit and scientific publication of the data in multiple articles illustrates the power of teens to use their lived experience to change the world. The TAC experience has provided a source of hope and inspiration for those involved as mentors as we observed the student's ingenuity and progress with awe. We would challenge other communities to empower adolescents to identify needs and capture their own experience to inform medical care and broader community solutions for complex stressors.

## Data Availability Statement

The original contributions presented in the study are included in the article/supplementary materials, further inquiries can be directed to the corresponding author/s.

## Ethics Statement

The studies involving human participants were reviewed and approved by LSU Heath Sciences Center—Shreveport. Written informed consent to participate in this study was provided by the participants' legal guardian/next of kin.

## Author Contributions

RL, JT, LA, PM, and JM contributed to the conception and design of the study. VA-Q performed the statistical analysis. PM, JT, and LA wrote sections of the article. KM, JP, and CS contributed to manuscript revision. All authors contributed to the article and approved the submitted version.

## Funding

This work was completed with the support of the Department of Psychiatry and Behavioral Medicine, the Institute for Childhood Resilience, the Louisiana Addiction Research Center, and the Department of Pharmacology, Toxicology, and Neuroscience at the Louisiana State University Health Sciences Center -Shreveport. It was also supported by the Community Foundation of Northwest Louisiana and Caddo Parish Schools.

## Conflict of Interest

The authors declare that the research was conducted in the absence of any commercial or financial relationships that could be construed as a potential conflict of interest.

## Publisher's Note

All claims expressed in this article are solely those of the authors and do not necessarily represent those of their affiliated organizations, or those of the publisher, the editors and the reviewers. Any product that may be evaluated in this article, or claim that may be made by its manufacturer, is not guaranteed or endorsed by the publisher.
